# Supplementary material for: Structural and functional characterization of the mitochondrial complex IV assembly factor Coa6
Source: Life Sci Alliance. 2019 Sep 12;2(5):e201900458. doi: 10.26508/lsa.201900458 (PMC6743065; doi:10.26508/lsa.201900458)
Supplement: Supplementary file 1 [file LSA-2019-00458_TableS1.docx]

Table S1. ^WT^Coa6 mass determined *via* MALDI-TOF following reduction and iodoacetamide labeling.

| Number of free Cysteines | Predicted Mass of ^WT^Coa6 upon alkylation (Da) | Determined mass of oxidized ^WT^Coa6 upon alkylation (Da) | Determined mass of reduced ^WT^Coa6 upon alkylation (Da) |
| --- | --- | --- | --- |
| No free Cysteines  (Fully oxidized) | 9839.07 | 9839***** | 9838.2 |
| 1 free Cysteine | 9897.07 | 9897 | 9896.3 |
| 2 Free Cysteines | 9955.07 |  | 9954.1***** |
| 3 free Cysteines | 10013.07 |  | 10012.6 |
| 4 free Cysteines  (Fully reduced) | 10071.07 |  | 10069.1 |

***** The dominant species in solution.
